# Comparison of Different Methods of Molecular Detection of *Erwinia amylovora* in Plant Material

**DOI:** 10.3390/cimb47121034

**Published:** 2025-12-11

**Authors:** Alexandr Pozharskiy, Valeriya Kostyukova, Gulnaz Nizamdinova, Dilyara Gritsenko

**Affiliations:** 1 Laboratory of Molecular Biology, Institute of Plant Biology and Biotechnology, Almaty 050040, Kazakhstan; 2 Research Center AgriBioTech, Almaty 050040, Kazakhstan; 3 Department of Molecular Biology and Genetics, Al Farabi Kazakh National University, Almaty 050040, Kazakhstan

**Keywords:** fire blight, molecular diagnostics, LAMP, real-time PCR, nanopore sequencing, 16S rRNA, *Malus domestica*, *Malus sieversii*

## Abstract

Fire blight, caused by *Erwinia amylovora*, is one of the most damaging bacterial diseases affecting apple production and the safety of wild *Malus sieversii* populations in Central Asia. Effective monitoring relies on accurate molecular diagnostics; however, comparative data on commonly used detection methods remain limited for the region. In this study, we evaluated the performance of three molecular assays—LAMP, real-time PCR, and targeted nanopore sequencing of a 16S rRNA gene fragment—using 124 plant samples exhibiting fire blight symptoms collected from 30 sites across Southern Kazakhstan and Kyrgyzstan. The results of LAMP, real-time PCR, and the amplification of 16S sequences were highly consistent with each other. Targeted 16S nanopore sequencing reliably identified *E. amylovora* in all PCR-positive samples, yielding high read counts and consistent species-level classification, although the analyzed 16S region provided limited resolution for intraspecies variation. Across sampling locations, abandoned orchards represented major reservoirs of infection compared to maintained orchards and wild populations. Our results confirm that all three approaches are robust tools for detecting *E. amylovora*. These findings support the importance of different molecular diagnostic methods to assist fire blight surveillance in the region.

## 1. Introduction

In Kazakhstan, fire blight caused by *Erwinia amylovora* is one of the most economically important and widespread diseases of apple trees, affecting both the yield quantity and quality. After its first detection in orchards in 2010, fire blight spread extensively throughout orchards in Kazakhstan and Kyrgyzstan [1]; moreover, infected trees have also been found in populations of the wild apple species *Malus sieversii* in the country [2]. Economic losses include the destruction of entire orchards; over 40 farms each year report new infection hotspots to phytosanitary authorities. The range of *E. amylovora* in Kazakhstan has been expanding annually, despite this pathogen being listed as a quarantine organism and under the supervision of phytosanitary authorities [3]. In recent years, cases of infection have been recorded not only in susceptible but also in resistant rootstocks and scions, as well as in wild apple trees [4]. The American wild apple germplasm originating from Kazakhstani populations showed 45% infected samples within a short period after the discontinuation of antibiotics that had been used for ten years [5], which indicates a widespread occurrence of *E. amylovora* among both cultivated and wild apple trees in the country. Meanwhile, large-scale studies aimed at assessing genetic diversity, identifying ribotypes, and characterizing strains of *E. amylovora* in apple species are still inadequate in the country. This highlights the importance of extensive monitoring of wild populations and apple orchards in the region to effectively control the spread of the infection. In this study, we tested three common molecular detection methods: LAMP, real-time PCR, and targeted nanopore sequencing of the 16S rRNA gene fragment to compare their applicability in terms of material and labor costs and to determine which approach is most effective for rapid and accurate diagnosis of *E. amylovora* in field and laboratory conditions. These three methods were tested on plant material with fire blight symptoms collected from various sites in southern Kazakhstan and Kyrgyzstan. In this paper, we present the results of evaluating the practical applicability of these methods and determining the optimal approach for diagnosing *E. amylovora* under different conditions.

## 2. Materials and Methods

**Figure 1:**
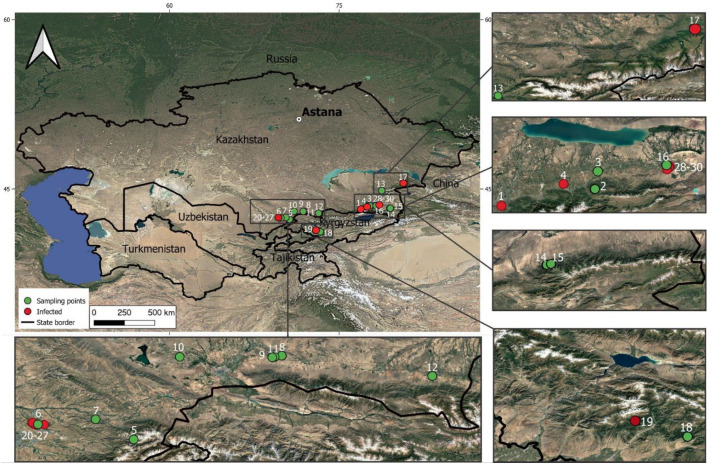
Map of the sampling locations of apple plant material infected by *E. amylovora*.

A search for apple plants affected by fire blight infection was conducted in the Turkestan, Almaty, Zhetysu, and Zhambyl regions, as well as in the Kyrgyz Republic, during the period of 2023–2025. The sampling locations included 30 sites, comprising wild populations of *Malus sieversii*, maintained orchards, and abandoned orchards of *M. domestica* (Table 1; Figure 1). The leaves from apple trees with different degrees of expression of the fire blight symptoms (dark necrotic spots), as well as the symptomless ones, were collected and stored at −80 °C until further use. Based on the expression of the symptoms, all samples were rated as 0 (symptomless), 1 (weak symptoms—occasional spots on the limited area of the leaves), or 2 (extensive leaf spots on the full area of the leaves).

DNA was extracted using the Plant/Fungi DNA Isolation Kit (Norgen Biotek, Thorold, ON, Canada) following the manufacturer’s protocol. From each sample, the leaf fragments of 100 mg containing the necrotic spots, if presented, have been used for DNA isolation. The integrity of the extracted nucleic acids was checked by electrophoresis on a 1.5% agarose gel, and their concentrations and A260/A280 ratios were measured with the NanoDrop spectrophotometer (Thermo Fisher Scientific, Waltham, MA, USA).

The LAMP and real-time PCR detection assays were conducted using primers recommended by the EPPO (standard 20/7 (3) [6] for *E. amylovora*) (Table 2). The LAMP reaction was performed using a commercial WarmStart Colorimetric LAMP 2× Master Mix with UDG kit (M1804L, New England BioLabs, Ipswich, MA, USA), in accordance with the manufacturer’s recommendations. The total reaction volume was 25 µL and included 12.5 µL of master mix with UDG, 2.5 µL of a mixture of specific primers for *Erwinia amylovora* (2 μM primers F3 and B3, 4 μM primers Loop-F and Loop-B, 16 μM primers FIP and BIP), 1 µL of isolated DNA (working dilution 20 ng/µL), and 9 µL of deionized water. The reactions were incubated in the SimpliAmp thermal cycler (Applied Biosystems, Thermo Fisher Scientific, Waltham, MA, USA) at the constant heating program 65 °C for 40 min, and the results were visually assessed based on the color change of the reaction mixture. Each reaction was repeated three times; the samples with at least two positive replicates have been considered positive for the reaction. When the visual resolution between the colors for negative and positive reaction was not clear, the agarose gel (1.5%) electrophoresis was additionally used to confirm the outcomes of LAMP reactions.

Real-time PCR was performed using standard *Taq* PCR Kit (New England Biolabs, Ipswich, MA, USA) following the EPPO protocol and the recommendations of the manufacturer. The reaction mix of the final volume of 20 µL contained 2 µL of 10× reaction buffer, 0.4 µL dNTP mix (10 mM), 0.4 µL of each primer and probe (10 µM), 0.2 µL *Taq*-polymerases, 1 µL isolated DNA (working dilution 20 ng/µL), and 15 µL deionized water. The assay was run on QuantStudio 5 Real-Time thermal cycler (Applied Biosystems, Thermo Fisher Scientific, Waltham, MA, USA) using the following program: 10 min at 95 °C, 40 cycles of 15 s at 95 °C, and 1 min at 60 °C. The samples were considered positive if the signal curve crossed the baseline before cycle 30 (Ct < 30).

For both tests, pure water (1 µL) and DNA extracted from the stock culture of *E. amylovora* (isolate E22 [7]) (1 µL of 5 ng/µL working solution) have been used as the negative and positive controls, respectively.

**Table 2:** Oligionucleotide primers used for detection of *E. amylovora*.

Assay	Primer	Sequence	Source
LAMP	Ea_Shin2018_F3	5′-ATA ATA AGA GAA TGG CGC TAT G-3′	EPPO 7/20 (3) [6]
	Ea_Shin2018_B3	5′-TCT ACA TCT CCA CCT TTG G-3′	
	Ea_Shin2018_FIP	5′-TAA TGA AGT TGA ATC TCA GGC ATG AGA AAA AAT CCA TTG TAA AAC CTT CG-3′	
	Ea_Shin2018_BIP	5′-GAT GGA TTG CTT AGT GAG CTC AGC CAA TCT CTC CAC AAC CG-3′	
	Ea_Shin2018_LoopF	5′-AAA GTT GTT TTC ATC CCA CGG A-3′	
Real-time PCR	Ams116F (forward)	5′-TCC CAC ATA CTG TGA ATC ATC CA-3	
	Ams189R (reverse)	5′-GGG TAT TTG CGC TAA TTT TAT TCG-3′	
	Ams141T (probe)	5′-**FAM**-CCA GAA TCT GGC CCG CGT ATA CCG-**TAMRA**-3′	
16S sequencing	Uni340F	5′-CCT ACG GGR BGC ASC AG-3′	[8]
	Uni806R	5′-GGA CTA CNN GGG TAT CTA AT-3′	

The targeted 16S sequencing was performed using the MinION MK1b device (Oxford Nanopore Technologies, Oxford, UK) and the SQK-NBD114-96 kit. The 16S rRNA gene fragment was amplified by PCR using universal prokaryotic primers Uni340F and Uni806R, according to Takai & Horikoshi, 2000 [8] (Table 2). The 25 µL reaction mixture contained 2.5 µL of 10× reaction buffer, 0.5 µL dNTP mix (10 mM), 0.5 µL of each primer (10 µM), 0.2 µL of *Taq*-polymerases, 2 µL of isolated DNA (working dilution 20 ng/µL), and 18.8 µL of deionized water. PCR conditions: initial denaturation at 94 °C for 3 min; 30 cycles of 94 °C for 30 s, 55 °C for 30 s, 72 °C for 1 min; and final elongation at 72 °C for 10 min. Amplicons of the expected length (about 500 bp) were verified in 1.5% agarose gel and DNA concentration was measured using a Qubit Flex fluorometer (Thermo Fisher Scientific, Waltham, MA, USA), after which the material was used for sequencing according to the manufacturer’s instructions. Basecalling and barcode demultiplexing were performed in Dorado v7.4.12 using a high-precision Q9 model, a minimum Q9 read quality threshold, and a barcode quality threshold of 60. Reads of lengths less than 200 bp were discarded. The obtained reads have been preliminarily identified using Kraken v. 1.1 [9] and then aligned against the rRNA gene sequences of *E. amylovora* (NR_041970.1) using BWA v. 0.7.17 tool [10], and the consensus sequences for each sample was assembled using SAMTools v. 1.19 [11]. The obtained consensus sequences have been tested using the NCBI BLAST online tool (https://blast.ncbi.nlm.nih.gov, accessed on 9 November 2025) to confirm the identification of *E. amylovora*. Furthermore, 16S were aligned along with the similar sequences from the NCBI GenBank database using MAFFT v. 7.453 [12], and the neighbor-joining tree with 1000 bootstrap replicates was calculated using MEGA v.11 [13] with the default model parameters.

## 3. Results and Discussion

A total of 124 samples were collected from 30 locations (Table 1). The observed symptoms were the necrotic spots on leaves. Of the total 124 collected samples, 72 were symptomless, 23 samples had weak symptoms rated as “1”, and 9 samples demonstrated stronger symptoms rated as “2”. No pattern of distribution of the samples with varying disease severity or absence across 30 sampling locations has been observed. All samples were successfully used to isolate DNA; the total concentration based on A260 index varied from 121.4 ng/μL to 402.0 ng/μL, with the average value of 249.8 ng/μL. The A260/A280 ratio varied from 1.69 to 2.01 and the A260/A230 ratio varied from 1.97 to 2.24. The electrophoresis confirmed the integrity of the isolated DNA with only slight smearing patterns present. No differences in quantity and quality of the isolated DNA were observed between symptomatic and symptomless samples.

Three identification methods based on different molecular approaches were selected. LAMP and real-time PCR methods were used as internationally recommended standards by EPPO [6]. 16S rRNA analysis was used to refine the identification, as discrepancies were observed between the LAMP and PCR results. In addition, targeted 16S rRNA sequencing allowed for detailed characterization of the bacterial pattern of the samples under investigation. The LAMP test was initially performed as the fastest and easiest method to interpret. Then, the samples were tested by real-time PCR. As a result of the testing, 26 out of 124 apple leaf samples tested positive using either of the methods employed (Table 3). Only 2 of 72 symptomless samples were tested positive by three tests and 1 sample was positive by LAMP but did not yield any PCR product. Fifteen of twenty-three samples with weak symptoms (“1”) and eight of nine samples with stronger symptoms (“2”) were tested positive by all three methods. LAMP and real-time PCR showed high agreement: only two samples were LAMP-negative while PCR was positive. The reactions for 16S amplification were consistent with the real-time PCR results.

**Figure 2:**
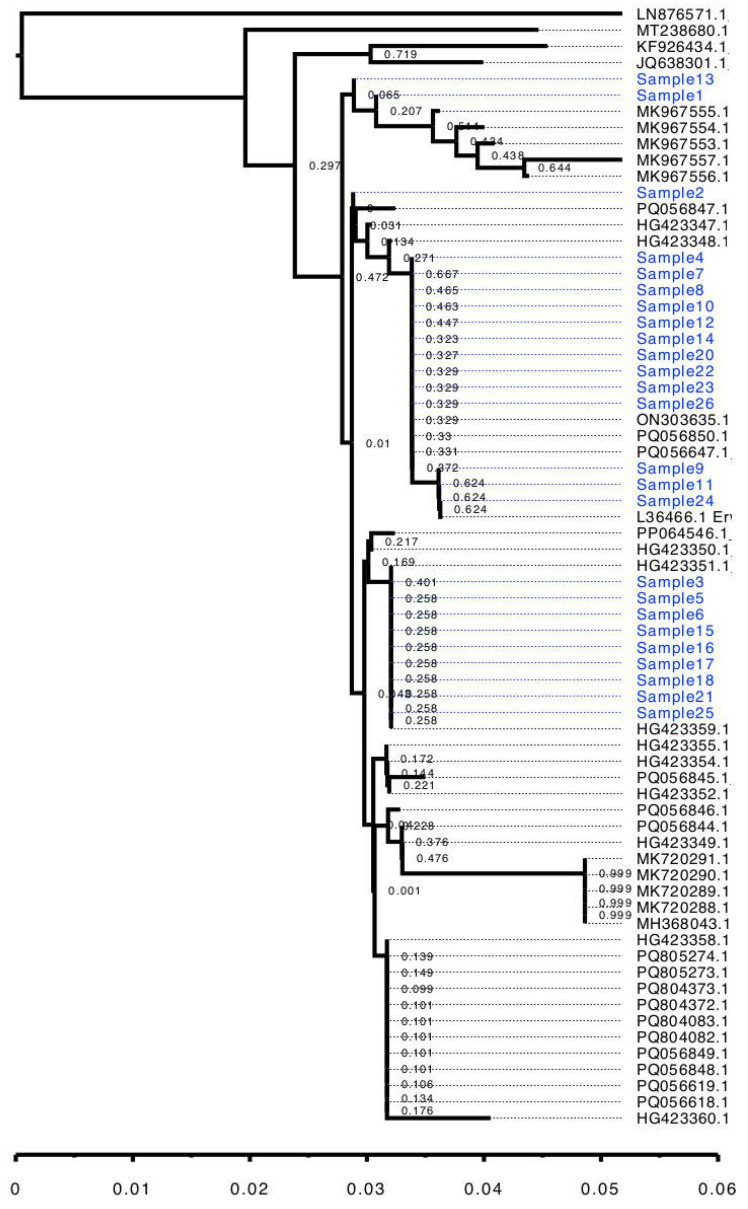
Neighbor-joining tree of 25 *Erwinia amylovora* 16S sequences. Sequences obtained in the present study shown blue.

The nanopore sequencing of 16S rRNA PCR products resulted between 10,842 and 13,900 reads per sample, and the percentage of reads specifically identified as belonging to *E. amylovora* ranged from 69% to 83%. The reads were successfully aligned against the reference rRNA gene of *E. amylovora* and, thus, sequences of the 16S fragment were assembled. The BLAST search identified all sequences as belonging to *E. amylovora* with high confidence (Table 3): for each sequence, between 86 and 104 hits corresponding to the target species were found, with an average identity percentage from 98.46% to 100%; no hits outside the *Erwinia* genus were identified. Therefore, 16S rRNA sequence analysis provided an unambiguous classification of the detected pathogen as *E. amylovora*. However, the subsequent phylogenetic analysis demonstrated that the analyzed 16S rRNA sequence region offers only limited information about within-species variability. As shown by Figure 2, the 25 obtained sequences were dispersed across multiple groups along with sequences from GenBank; the tree pattern did not show any correlation to the geographic distribution of the samples. The main nodes of the tree also exhibited low bootstrap support values, which are related to the short length of the sequences and their low variability [14]. Thus, although the used 16S rRNA sequence provides good species identification, its informational content is insufficient to draw conclusions at the intraspecies level.

As a result of the testing, we conclude that all three assays—LAMP, real-time PCR, and targeted 16S rRNA sequencing—are reliable for identifying *E. amylovora* in plant samples. The data available to date allow comparisons of different aspects of these methods (Table 4). LAMP and PCR proved to be highly effective tools, offering high sensitivity and specificity in fire blight diagnostics [15]. The main advantages of LAMP are speed and low equipment and labor requirements: only isothermal amplification equipment is used, making the method the most accessible and the fastest [16]. Real-time PCR remains the gold standard of molecular diagnostics, as the combination of specific primers and probes increases sensitivity and specificity [17]. However, it requires a specialized thermal cycler and more expensive equipment, making it the most costly of the three methods. Finally, 16S sequencing is more costly and labor-intensive but provides the most reliable identification based on the between-species sequence variation. The sequence-based analysis also helps to look further on the sequence variability and directly compare the obtained sequences to the previous data available in the databases using phylogenetic methods (Figure 2). The emerging use of nanopore sequencing makes it more convenient. Nanopore sequencing is a versatile tool for both whole genome and targeted sequencing across a wide range of pathogens [18]. The cost of nanopore sequencing is higher than LAMP due to consumables, but lower than real-time PCR. An important property of nanopores is their ability to individually read and process DNA molecules, making it possible to separate DNA from different sources and thus overcome potential DNA contamination, such as admixture of PCR products from the pathogen and its host, which is an important problem making the traditional Sanger sequencing more difficult. Table 4 presents a comparison of all three methods.

A comparison of the methods shows that differences in their limits of detection determine their practical roles. LAMP has the lowest sensitivity (LOD 10^4^–10^5^ cells/mL), which is reflected in its reduced diagnostic sensitivity. Nevertheless, its speed, simplicity, and minimal equipment requirements make it an effective tool for preliminary screening and field surveys, where rapid turnaround is critical. Real-time PCR remains the most sensitive and reliable method (LOD 5 × 10^2^–5 × 10^3^ cells/mL), providing high diagnostic sensitivity and specificity. This makes PCR the optimal choice for confirmatory diagnostics and official phytosanitary testing, although its high cost and the need for stationary equipment limit its use outside the laboratory. Targeted 16S/HTS sequencing occupies a distinct position: with sufficient read depth, it offers high sensitivity and enables assessment of the microbial context, which is important for ambiguous or complex samples. Its limitations include the risk of errors at low read numbers and substantially higher analytical labor. In practical terms, HTS is justified only when standard methods produce contradictory results or when extended information about the sample’s microbiome is required. The use of various molecular detection methods, such as LAMP and real-time PCR, allowed us to determine the presence of *E. amylovora* in various apple tree populations and orchards in southern Kazakhstan and Kyrgyzstan. These preliminary results indicate the direction for further studies, e.g., based on the regular monitoring of populations. The abandoned orchards were found to be hotspots for the presence of *E. amylovora*, compared to the functioning orchards under phytosanitary control, as well as wild populations of *M. sieversii*. The results from the three assays were highly consistent, making any of these methods or their combinations suitable tools for detection and monitoring of fire blight in the country.

## 4. Conclusions

We compared three commonly used molecular assays—LAMP, real-time PCR, and targeted nanopore sequencing of the 16S rRNA fragment—for detecting *Erwinia amylovora* in symptomatic apple plant material collected from Southern Kazakhstan and Kyrgyzstan. All three methods proved reliable for precise species identification of the fire blight pathogen, showing a high level of agreement among them. LAMP offered clear advantages as a rapid, cost-effective, and equipment-minimal diagnostic tool suitable for field or low-resource settings. Real-time PCR remained the most sensitive and specific laboratory standard, while 16S nanopore sequencing provided unambiguous species-level identification and additional confirmation of diagnostic results, although its ability to resolve intraspecies diversity was limited. Integrating these complementary approaches allowed for effective screening across various sampling sites and revealed abandoned orchards as major reservoirs of infection compared with maintained orchards and wild *Malus sieversii* populations. The findings underscore the importance of ongoing monitoring and early detection to curb the spread of fire blight in the region. Using a combination of rapid assays and high-precision sequencing will enhance phytosanitary surveillance programs and support timely management strategies to protect both cultivated and wild apple genetic resources.

## Figures and Tables

**Table 1:** Samplinglocations of apple plant material. The populations where samples with *Erwinia amylovora* infection have been detected are shown in bold.

Number	Region	Location (Coordinates, °)	Type	Number of Samples
**1**	**Almaty**	**43.226801; 76.916254**	**Orchard**	**3**
2	Almaty	N/A (Shelek village)	Orchard	5
3	Almaty	43.510556402; 77.702780175	Orchard	4
**4**	**Almaty**	**43.406389741; 77.423057951**	**Orchard**	**5**
5	Turkestan	42.316667657; 70.616669068	Orchard	4
6	Turkestan	42.44023; 69.78490	Orchard	3
7	Turkestan	42.490000994; 70.290002409	Orchard	4
8	Zhambyl	43.035192625; 71.888579086	Orchard	2
9	Zhambyl	43.022715960; 71.817559086	Orchard	6
10	Zhambyl	43.026072641; 71.013099090	Orchard	3
11	Zhambyl	43.022852627; 71.823817419	Orchard	5
12	Zhambyl	42.863334271; 73.174169072	Orchard	3
13	Zhetysu	44.863056365; 78.764169105	Orchard	5
14	Almaty	43.293056816; 79.484169376	Wild population	6
15	Almaty	43.305278810; 79.751391374	Wild population	4
16	Almaty	43.364244848; 77.680407392	Wild population	3
**17**	**Zhetysu**	**45.517460764; 80.722242446**	**Wild population**	**5**
18	Kyrgyz Republic	41.210000953; 73.336669017	Wild population	4
**19**	**Kyrghyz Republic**	**41.333334292; 72.933335690**	**Wild population**	**5**
**20**	**Turkestan**	**42.44863821; 69.78856815**	**Abandoned orchard**	**5**
**21**	**Turkestan**	**42.44779721; 69.79035526**	**Abandoned orchard**	**6**
**22**	**Turkestan**	**42.44968945; 69.78657079**	**Abandoned orchard**	**4**
**23**	**Turkestan**	**42.44874333; 69.76197175**	**Abandoned orchard**	**3**
**24**	**Turkestan**	**42.46041211; 69.74557239**	**Abandoned orchard**	**3**
**25**	**Turkestan**	**42.46230434; 69.75187984**	**Abandoned orchard**	**5**
**26**	**Turkestan**	**42.44522167; 69.84775302**	**Abandoned orchard**	**4**
**27**	**Turkestan**	**42.44401275; 69.85153749**	**Abandoned orchard**	**4**
**28**	**Almaty**	**43.55170546; 78.28706375**	**Abandoned orchard**	**3**
**29**	**Almaty**	**43.55202083; 78.28900855**	**Abandoned orchard**	**5**
**30**	**Almaty**	**43.54077255; 78.28506639**	**Abandoned orchard**	**3**
			Total:	124

**Table 3:** Results of apple plant material testing for *Erwinia amylovora* using LAMP, real-time PCR, and targeted 16S rRNA sequencing. Only positive samples are shown.

Sample Number	Sampling Population	Symptoms ^1^	LAMP (Positive/Negative)	PCR (Ct)	16S ^2^	Sample Number	Sampling Population	Symptoms ^1^	LAMP (Positive/Negative)	PCR (Ct)	16S ^2^
1	1	1	+	15.7951	101; 98.98%	14	24	1	+	13.1110	100; 100%
2	1	0	+	18.1443	90; 98.85%	15	24	2	+	14.1351	86; 100%
3	1	1	+	15.9387	97; 100%	16	25	2	+	15.8117	86; 100%
4	4	1	+	13.9387	100; 100%	17	26	1	+	17.7767	97; 100%
5	17	1	+	14.5635	97; 100%	18	26	1	−	19.8467	97; 100%
6	29	2	+	14.8362	97; 100%	19	26	0	+	-	-
7	20	1	+	17.3393	100; 100%	20	27	2	+	15.1253	100; 100%
8	20	2	+	18.1095	100; 100%	21	28	1	+	16.5928	97; 100%
9	20	0	+	15.2245	100; 100%	22	29	1	+	12.6914	104; 100%
10	21	2	+	14.1580	100; 100%	23	29	1	+	17.8952	100; 100%
11	21	2	+	17.3028	101; 100%	24	29	1	+	17.3102	100; 100%
12	22	1	+	16.2087	100; 100%	25	30	1	+	16.5955	86; 100%
13	23	1	−	13.3125	99; 98.46%	26	30	2	+	14.8070	102; 100%

^1^ Symptoms rated as described above in the Section 2. ^2^ Number of the BLAST hits for 16S sequences identified as *Erwinia amylovora* and the average percentage of identity of the sequences aligned by BLAST.

**Table 4:** Comparison of LAMP, real-time PCR, and targeted 16S sequencing for detection of *Erwinia amylovora*.

Criterion	LAMP	Real-Time PCR	16S/HTS (Target Sequencing)
Analytical sensitivity (LOD)	10^4^–10^5^ cells/mL (for plant samples) [6,19]	5 × 10^2^–5 × 10^3^ cells/mL (for plant samples) [6,19]	Depending on assay; not reported for *E. amylovora*
Diagnostic sensitivity	65% [6,19]	84% [6,19]	High with sufficient number of reads; risk of confusion with closely related genera at low depth [20]
Diagnostic specificity	98% [6,19]	97% [6,19]	
Analysis time	20–60 min	1.5–2.5 h	8–12 h
Labor intensity	Low: minimal sample preparation, isothermal mode	Intermediate: DNA extraction, PCR, interpretation of analysis curves	High: library preparation and data analysis
Equipment requirements	Only a thermoblock or portable heater	RT-PCR thermal cycler	Sequencer + infrastructure for data analysis
Test cost	The cheapest method	The most expensive due to the cost of equipment	Average cost when using the ONT platform
Practical applicability	Rapid screening, field tests, nurseries, express diagnostics	Confirmatory method, quantitative determination, official phytosanitary inspections	Verification of results, disputed samples, analysis of complex matrices

## Data Availability

The original contributions presented in this study are included in the article/Supplementary Materials. Further inquiries can be directed to the corresponding author.

## Supplementary Materials

The following supporting information can be downloaded at: https://www.mdpi.com/article/10.3390/cimb47121034/s1.
